# Proteomic analysis of surface proteins of *Trichinella spiralis* muscle larvae by two-dimensional gel electrophoresis and mass spectrometry

**DOI:** 10.1186/1756-3305-6-355

**Published:** 2013-12-16

**Authors:** Jing Cui, Ruo Dan Liu, Li Wang, Xi Zhang, Peng Jiang, Ming Yuan Liu, Zhong Quan Wang

**Affiliations:** 1 Department of Parasitology, Medical College, Zhengzhou University, 40 Daxue Road, Zhengzhou 450052, P. R. China; 2 Key Lab of Zoonosis Research, Ministry of Education, Institute of Zoonosis, Jilin University, Changchun 130062, P. R. China

**Keywords:** *T. spiralis*, Muscle larvae, Surface proteins, Mass spectrometry

## Abstract

**Background:**

*Trichinella spiralis* is a zoonotic tissue-dwelling parasitic nematode that infects humans and other mammals. Its surface proteins are recognized as antigenic in many infected hosts, being directly exposed to the host’s immune system and are the main target antigens that induce the immune responses. The larval surface proteins may also interact with intestinal epithelial cells and may play an important role in the invasion and development process of *T. spiralis*. The purpose of this study was to analyze and characterize the surface proteins of *T. spiralis* muscle larvae by two-dimensional gel electrophoresis (2-DE) and mass spectrometry.

**Methods:**

The surface proteins of *T. spiralis* muscle larvae were stripped from the cuticle of live larvae by the cetyltrimethylammonium bromide (CTAB) and sodium deoxycholate. The surface protein stripping was examined by an immunofluorescent test (IFT). The surface proteins were analyzed by SDS-PAGE and Western blotting, and then identified by 2-DE and MALDI-TOF/TOF mass spectrometry analysis.

**Results:**

The IFT results showed that the surface proteins-stripped larvae were not recognized by sera of mice immunized with surface antigens. Western blotting showed 7 of 12 protein bands of the surface proteins were recognized by mouse infection sera at 18 dpi and at 42 dpi. The 2-DE results showed that a total of approximately 33 proteins spots were detected with molecular weights varying from 10 to 66 kDa and isoelectric point (pI) from 4 to 7. Twenty-seven of 33 protein spots were identified and characterized to correlate with 15 different proteins. Out of the 14 proteins identified as *T. spiralis* proteins, 5 proteins (partial P49 antigen, deoxyribonuclease II family protein, two serine proteases, and serine proteinase) had catalytic and hydrolase activity. All of these 5 proteins were also associated with metabolic processes and 2 of the five proteins were associated with cellular processes.

**Conclusions:**

In this study, *T. spiralis* muscle larval surface proteins have been identified, which will provide useful information to elucidate the host-parasite interaction, identify the invasion-related proteins, early diagnostic antigens and the targets for a vaccine.

## Background

*Trichinella spiralis* is a tissue-dwelling parasitic nematode infecting many kinds of carnivores and omnivores, and is the main causative agent of trichinellosis. Humans acquire the disease by the ingestion of raw or insufficiently cooked meat containing the *T. spiralis* infective larvae [1]. Once ingested, muscle larvae are released from their capsules in the stomach by the digestive enzymes. Then, the muscle larvae invade, occupy and migrate through intestinal epithelium cells where they undergo four molts to emerge as sexually mature adults [2]. The establishment of *T. spiralis* in this habitat is the key step in which the larvae infect the hosts. With regard to the intestinal stage of infection, it has been suggested that proteases participate in intestinal invasion by *T. spiralis*[3,4]. Although it has been known for many years that *T. spiralis* larvae invade the intestinal epithelium and the *in vitro* model of epithelial invasion by the larvae has been developed [5,6], the mechanisms by which *T. spiralis* infective larvae recognize, invade, and migrate within the intestinal epithelium are unknown.

Previous studies had showed that the cuticle surface of parasitic nematodes is recognized as antigenic in many infected hosts [7,8]. In a number of experimental systems antibodies are produced against surface molecules and mediate antibody dependent cell mediated cytotoxic reactions [9]. *T. spiralis* surface proteins are directly exposed to the host’s immune system, are the main target antigens which induce the immune responses, and may play an important role in the invasion and development process of *T. spiralis* larvae. There has been special interest to study the *T. spiralis* surface proteins that interact at the interface between the parasite and the host to modify the environment, either by modulating the host immune response or even host cell gene expression, to ensure parasite invasion, development and survival [10,11]. The surface proteins may also be involved in the larvae-nurse cell complex formation and maintenance during the muscular stage of the infection. Therefore, analysis of *T. spiralis* muscle larval surface proteins and characterization of their molecular function and biological process could provide important information to elucidate the mechanism of parasite invasion and possibly identify invasion-related proteins, early diagnostic antigens and potential targets for a vaccine.

Recently, proteomic approaches are being used to complement genetic studies on *T. spiralis*[12]. As effective tools for proteomics, the two-dimensional electrophoresis (2-DE) combined with mass spectrometry (MS) has been widely used to characterize the differential expression profiles of different species of *Trichinella* spp. [13-17]. Because the excretory- secretory (ES) proteins were easily prepared by the *in vitro* cultivation of *Trichinella* muscle larvae, the ES proteins were usually analyzed by utilizing 2-DE techniques [18,19]. To our knowledge, no surface proteins of *T. spiralis* muscle larvae have been analyzed and identified by 2-DE and mass spectrometry.

In this study, the surface proteins of *T. spiralis* muscle larvae were firstly stripped and analyzed, then identified and characterized by the 2-DE combined with Matrix-assisted laser desorption ionization (MALDI)-time-of-flight (TOF)/TOF-MS approach. It is therefore of fundamental importance for further studies of the surface protein functions on the invasion, survival, and development of *T. spiralis* and the early diagnostic markers for trichinellosis.

## Methods

### Parasite and experimental animals

*Trichinella spiralis* isolate (ISS534) used in this study was obtained from a domestic pig in Nanyang city of Henan Province, China. The isolate was maintained by serial passages in Kunming mice in our laboratory. Six-week-old male Kunming mice were obtained from the Experimental Animal Center of Henan Province (Zhengzhou, China). The mice were maintained under specific pathogen-free conditions with sterilized food and water.

### Collection of infection sera

BALB/c mice were orally infected with 300 muscle larvae/mouse and the serum samples from the infected mice were collected as described previously [20]. About 100 μl of tail vein blood was collected daily from each mouse before infection and during 14–21 days post infection (dpi), respectively. When the forty infected mice were sacrificed at 42 dpi by deep ether anesthesia, their serum samples were also collected. Anti-*Trichinella* IgG antibodies in sera from infected mice at 14–21 dpi were assayed by ELISA and Western blot. The specific antibodies were firstly detected at 18 dpi and persisted to 42 dpi by the above-mentioned two methods, and then these sera collected at 18 dpi and 42 dpi were used to detect the following surface proteins.

### Preparation of surface, ES and somatic proteins

The muscle larvae were recovered from the mice infected with 300 *T. spiralis* infective larvae at 42 dpi by artificial digestion of carcasses with 1% pepsin (1:3,000) and 1% hydrochloric acid as described previously [20,21]. Muscle larval surface proteins were prepared as the previously described method with some modification [8,22]. Briefly, the live muscle larvae were cultured in phosphate-buffered saline (PBS; pH 7.4, 1/15 mol/L) contained 0.25% cetyltrimethylammonium bromide (CTAB;Sigma, USA) and 2% sodium deoxycholate (Sigma, USA) at 37°C for 2.5 h. The supernatant was obtained by centrifugation at 4°C, 11,000 × g for 20 min, and dialyzed against deionized water at 4°C for 2 days.

The ES proteins of *T. spiralis* muscle larvae were prepared as described previously [23]. In brief, after washing thoroughly in sterile saline, the larvae were again washed four times in serum-free RPMI-1640 medium supplemented with 100 U penicillin/ml and 100 μg streptomycin/ml. The larvae were incubated in the same medium at concentration of 5 000 worms/ml for 18 h at 37°C in 5% CO_2_. After incubation, the media containing the ES products were filtered through a 0.2 μm membrane into a 50-ml conical tube, then centrifuged at 4°C, 15,000 × g for 30 min. The supernatant was dialyzed against deionized water at 4°C for 2 days.

The supernatant containing surface or ES proteins were concentrated by a vacuum concentration and freeze drying (Heto Mxi-Dry-Lyo, Denmark). The protein concentration of surface proteins (4.62 mg/ml) or ES proteins (1.26 mg/ml) was determined by the method described by Bradford [24]. The surface or ES proteins were aliquoted and stored at -20°C before use.

Somatic proteins were prepared from *T. spiralis* muscle larvae resuspended in deionized water. The suspension was submitted to 5 cycles of freezing-thawing. The larvae were homogenized on ice in a glass tissue grinder. After this, the larval fragments were further homogenized with ultrasonication (99 times 3-s cycle, 100 W, 0°C). The supernatant was collected after centrifugation at 15,000 g for 1 h at 4°C. The protein concentration of somatic proteins (1.25 mg/ml) was determined by the method described by Bradford [24].

### Generation of mouse immune sera to surface proteins

Ten male BALB/c mice were used in this study. Pre-immune serum samples were collected by tail bleeding 2 days prior to the first immunization. BALB/c mice were subcutaneously immunized with 20 μg of surface proteins emulsified with complete Freund’s adjuvant (FCA), followed by three boosts with the same amount of protein emulsified with incomplete FCA at 10-day intervals. Seven days after the last boost, mice were bled and the sera were collected [4].

### Immunofluorescent test (IFT)

IFT was used to detect the stripped surface proteins of *T. spiralis* muscle larvae. The normal and surface proteins-stripped muscle larvae were collected respectively, and were fixed in 4% paraformaldehyde. The whole muscle larvae were blocked with 5% normal goat serum in PBS and then incubated in a moist chamber at 37°C for 1 h with a 1:10 dilution of immune and normal sera. After washing three times in PBS, the larvae were incubated with a 1:20 dilution of FITC-labeled goat anti-mouse IgG (Santa Cruz, USA), washed five times in PBS, and examined under a fluorescent microscope (Olympus, Japan) [25].

### SDS-PAGE and Western blotting

Protein samples including surface, ES or somatic proteins were diluted with loading buffer (250 mM Tris–HCl pH 6.8, 50% glycerol, 10% SDS, 5% 2-mercaptoethanol, 0.5% bromophenol blue) up to a concentration of 15 μg/lane. After cooling, the proteins were separated by SDS-PAGE on 12% acrylamide separating gel and 5% acrylamide stacking gels (83 × 73 × 1.0 mm) in a Mini-PROTEAN 3 Cell electrophoresis unit (Bio-Rad, USA) at 120 V for 2.5 h [26]. After electrophoresis, the gel was stained with 0.25% Coomassie brilliant blue R-250 (Sigma, USA) for 4 h, and then bleached with the eluate (100 mL acetic acid, 50 mL ethanol, 850 mL dH_2_O). A second gel was prepared with the above-mentioned proteins.

After electrophoresis, proteins were transferred to nitrocellulose membrane (Millipore, USA). After blotting, the membranes were stained with Ponceau S to verify transfer and to locate the protein marker and cut into strips. Each strip was blocked with 5% skimmed milk in Tris-Buffered Saline with Tween-20 (TBST) at 37°C for 2 h, and incubated overnight with 1:100 dilutions of the different mouse sera. After washing, the strips were incubated at 37°C for 1 h with HRP-conjugated goat anti-mouse IgG (1:5000 dilution; Southern Biotechnology, USA), and finally with 3, 30-diaminobenzidine tetrahydrochloride (DAB; Sigma). The reaction was finally stopped by washing the strips with distilled water.

### 2-DE and image analysis

The surface antigens were precipitated using trichloroacetic acid (TCA) and acetone as for the previously described method with some modifications [27]. Briefly, the sample was suspended in 10% TCA in acetone with 20 mM DTT at -20°C for 2 h. After centrifugation at 15,000 g at 4°C for 15 min, the pellet was resuspended in cold acetone containing 20 mM DTT and washed three times. The final pellet was air-dried. The 2-DE was performed as previously described [15]. In brief, the pellet was suspended in rehydration buffer [7 M urea, 2 M thiourea, 4% CHAPS, 65 mM DTT, 0.2% IPG buffer (pH 3–10) and 0.001% bromophenol blue], containing 800 μg of the protein samples in a total volume of 500 μl and centrifuged at 12,000 g for 10 min at room temperature to remove the insoluble materials. The supernatant was loaded onto 24 cm pH 4–7 immobilized pH gradient (IPG) strips (Bio-Rad, USA) by over-night re-swelling and separated by isoelectric focusing (IEF) using a Protean IEF Cell (Bio-Rad, USA). IEF was performed using a Protean IEF Cell at 20°C as follows: S1: 250 V, 30 min; S2: 500 V, 30 min; S3: 1000 V, 1 h; S4: 10 000 V, 5 h; and S5: 10 000 V, 60 000 Vh (using a limit of 50 μA/strip). After IEF, the IPG strips were equilibrated sequentially, first in equilibration buffer (6 M urea, 0.375 M Tris–HCl pH 8.8, 2% SDS and 20% glycerol) containing 2% dithiothreitol, then in equilibration buffer containing 2.5% iodoacetamide. The second dimension was performed on 12% SDS-PAGE using a Mini Protean cell (Bio-Rad, USA). Proteins were separated for 30 min at 16 mA and then at 24 mA until the dye front reached the bottom of the gel at 16°C. After 2-DE, proteins were stained with Coomassie blue R-250 for proteomic analysis as previously described [26]. The gel was scanned using ImageScanner (GE healthcare, USA). Spot detection and spot matching were performed by using Image Master 2D Platinum 6.0 (GE healthcare, USA). Three replicates were run for the sample. Only those protein spots that were clearly observed in three independent experiments were chosen for further analysis.

### 2-DE gel excision and tryptic digestion

2-DE gel electrophoresis protein spots were prepared for MALDI-TOF/TOF-MS analysis according to standard protocols [28]. Thirty-three spots were excised manually from the Coomassie blue-stained gels. The excised gel pieces carrying the spots were placed in a tube, destained for 20 min in 200 mmol/L NH_4_HCO_3_/30% acetonitrile and then lyophilized. All the lyophilized samples were digested overnight at 37°C with 12.5 ng/ml trypsin in 25 mmol/L NH_4_HCO_3_. The peptides were extracted three times with 60% ACN/0.1% trifluoroacetic acid (TFA). The extracts were pooled and dried completely by centrifugal lyophilization.

### Protein identification by MALDI-TOF/TOF-MS

The resulting peptides from the above extraction were analyzed by MALDI-TOF/TOF-MS. The procedures were performed as described previously [16]. Briefly, The purified tryptic peptide samples were spotted onto stainless steel sample target plates and mixed (1:1 ratio) with a matrix consisting of a saturated solution of a-cyano-4-hydroxy-trans-cinnamic acid in 50% acetonitrile-1% TFA. Peptide mass spectra were obtained on an Applied Biosystem Sciex 4800 MALDI-TOF⁄TOF mass spectrometer (Applied Biosystems, USA). Data were acquired using a CalMix5 standard to calibrate the instrument (ABI4700 Calibration Mixture). The MS spectra were recorded in reflector mode in a mass range from 800 to 4000 with a focus mass of 2000. For MS/MS spectra, up to 10 of the most abundant precursor ions per sample were selected as precursors for MS/MS acquisition, excluding the trypsin autolysis peaks and the matrix ion signals. In MS/MS positive ion mode, for one main MS spectrum, 50 subspectra with 50 shots per subspectrum were accumulated using a random search pattern. Collision energy was 2 kV, collision gas was air, and default calibration was set by using the Glu1-Fibrino-peptide B ([M + H] + 1, 570.6696) spotted onto Cal 7 positions of the MALDI target. Combined peptide mass fingerprinting (PMF) and MS/MS queries were performed by using the MASCOT search engine 2.2 (Matrix Science, UK) and submitted to MASCOT Sequence Query server (http://www.matrixscience.com) for identification against nonredundant NCBI database (http://www.ncbi.nlm.nih.gov/BLAST) with the following parameter settings: 100 ppm mass accuracy, trypsin cleavage (one missed cleavage allowed), carbamidomethylation set as fixed modification, oxidation of methionine was allowed as variable modification, and MS/MS fragment tolerance was set to 0.4 Da. The criteria for successfully identified proteins were as follows: ion score confidence index (CI) for peptide mass fingerprint and MS/MS data was ≥95%.

### Ethics statement

All animals were treated in strict accordance to the National Guidelines for Experimental Animal Welfare (MOST of People’s Republic of China, 2006). The protocols of the animal experiments reported herein were approved by The Life Science Ethics Committee of Zhengzhou University. All efforts were made to minimize animal suffering during the course of these studies.

## Results

### Examination of the larval surface protein stripping by IFT

**Figure 1:**
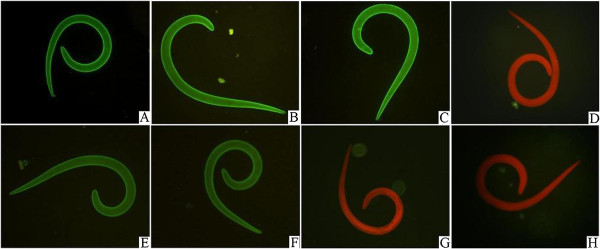
**Immunofluorescent staining of *T. spiralis *muscle larvae.** The cuticle surface of normal whole larvae were recognized by sera of mice infected with *T. spiralis* at 42 dpi **(A)** and 18 dpi **(B)**, sera of mice immunized with *T. spiralis* surface antigens **(C)**, but not by normal mouse sera **(D)**; The surface proteins-stripped larvae had week positive reaction with sera of mice infected with *T. spiralis* at 42 dpi **(E)** and at 18 dpi **(F)**, but they were not recognized by sera of immunized with surface antigens **(G)** and normal mouse sera **(H)**.

The IFT results were shown in Figure 1. The intense fluorescent staining at the cuticle surface of the normal whole larvae was found when the sera of mice infected with *T. spiralis* at 42 dpi as well as 18 dpi, and sera of mice immunized with surface antigens were used, but the larvae had negative reaction with sera of normal mice. The surface proteins-stripped larvae still had a weak positive reaction with sera of mice infected with *T. spiralis* at 42 dpi and 18 dpi, but they had negative reaction with sera of immunized with surface antigens and normal mouse sera. The results of IFT indicated that surface proteins of muscle larvae were successfully stripped.

### Analysis of the surface proteins by SDS-PAGE and Western blotting

**Figure 2:**
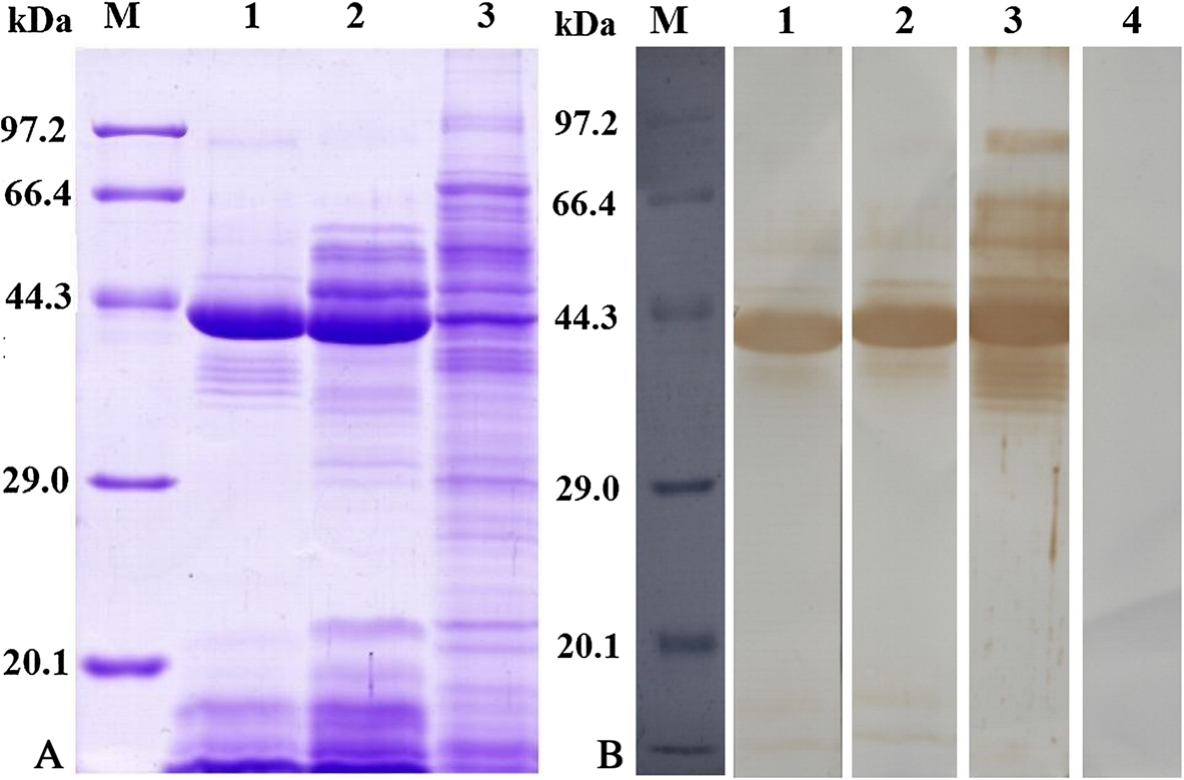
**Analysis of surface proteins from *T. spiralis *muscle larvae. (A)** SDS-PAGE analysis of surface proteins. Protein samples were separated in 12% polyacrylamide gels under reducing conditions, and the gels were stained with Colloidal Coomassie blue G-250. M, low molecular weight protein marker; Lane 1, surface proteins; Lane 2, ES proteins; Lane 3, somatic proteins from whole larvae. **(B)** Western blotting of surface proteins. The surface proteins were recognize were recognized by sera from mice infected with *T. spiralis* at 18 dpi (Lane 1) and at 42 dpi (Lane 2) and sera from mice immunized with surface proteins (Lane 3), not by sera from normal mice (Lane 4).

The results of Coomassie blue stained SDS-PAGE gels of surface proteins from *T. spiralis* muscle larvae are shown in Figure 2A. The surface proteins had 12 protein bands with a molecular weight of 92, 53, 47, 42, 39, 38, 37, 36, 35, 21, 18 and 16 kDa. We found that 7 protein bands (42, 39, 36, 35, 21, 18 and 16 kDa) appeared in both surface and ES proteins, and 7 protein bands (42, 39, 38, 35, 21, 18 and 16 kDa) appeared in both surface and somatic proteins. The results of Western blotting of surface proteins are shown in Figure 2B. Seven protein bands (53, 47, 42, 39, 38, 18 and 16 kDa) of surface proteins were recognized by mouse infection sera at 18 dpi and at 42 dpi. All 12 proteins of surface proteins were recognized by sera from mice immunized with surface proteins.

### 2-DE analysis of surface proteins from *T. spiralis* muscle larvae

**Figure 3:**
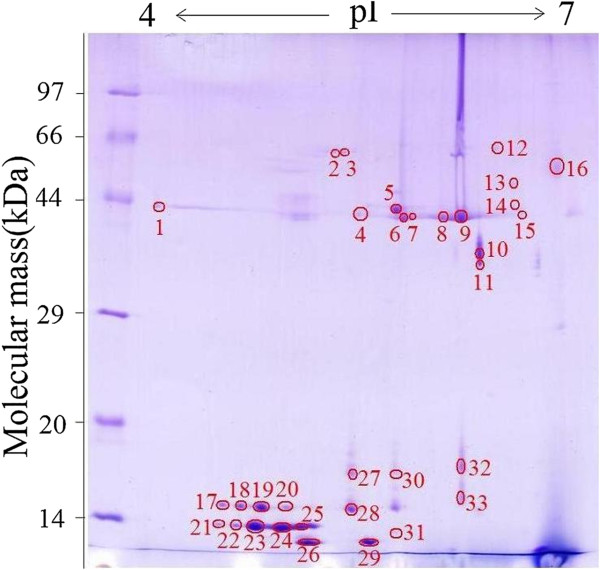
**2-DE analysis of *T. spiralis *muscle larval surface proteins.** 2-DE gel of surface proteins separated in the first dimension in the pH range 4–7 and then in the second dimension on a 12% polyacrylamide gel. The gel was stained with Coomassie blue R-250, molecular weight standard is on the left, and pI values are indicated. Protein spots selected for further analysis are numbered.

The surface proteins of *T. spiralis* muscle larvae were separated on a 2-DE gel covering a pH 4–7 nonlinear, and the protein spots were visualized following Coomasie R-250 staining (Figure 3). A total of approximately 33 spots were detected on the Coomassie blue stained 2-DE gels, with molecular weights (MW) varying from 10 to 66 kDa and pI from 4 to 7. Major protein spots were located in the acidic range (pH 4–6) migrating at 30–60 kDa and 10–20 kDa. The 2-DE was repeated three times, and the patterns were highly reproducible.

### Identification of proteins by MALDI-TOF/TOF-MS

Twenty-seven of 33 protein spots were identified and characterized to correlate with 15 different proteins. Fourteen out of the 15 different proteins identified were *T. spiralis* proteins. The results of protein identification are shown in Table 1. Several different protein spots were identified as the same proteins by MALDI-TOF/TOF-MS, such as spots 4, 6, 7, 9 and 15 were identified as deoxyribonuclease II family protein. Spots 17, 19, 20, 23, 24, and 25 were identified as the same conserved hypothetical protein.

**Table 1:** **Identification of *T. spiralis *** **muscle larval surface protein by MALDI-TOF/TOF-MS**

**Spot no.**	**Protein name**	**Accession no.**	**Theoretical Mr/pI**^**a**^	**MOWSE score**	**Coverage (%)**	**No. matched peptides**	***p*-value**
1	partial P49 antigen	gi|162542	34.5/5.23	80	15	3	0.00032
2	Not identified	-	-	-	-	-	-
3	hypothetical protein Tsp_08444	gi|339247637	18.9/9.70	62	57	7	0.023
4	deoxyribonuclease II family protein	gi|339241449	38.1/5.95	98	10	2	6.8e-006
5	serine protease	gi|168805931	35.7/5.97	245	18	4	1.1e-020
6	deoxyribonuclease II family protein	gi|339241449	38.1/5.95	382	22	6	2.1e-034
7	deoxyribonuclease II family protein	gi|339241449	38.1/5.95	315	22	6	1.1e-027
8	Not identified	-	-	-	-	-	-
9	deoxyribonuclease II family protein	gi|339241449	38.1/5.95	447	28	7	6.6e-041
10	ps73f07.y1 *T. spiralis* adult pAMP1 v1 *T. spiralis* cDNA 5′, mRNA sequence	gi|21410467	22.3/4.83	371	39	9	2e-032
11	ps97f02.y1 *T. spiralis* adult pAMP1 v1 *T. spiralis* cDNA 5′, mRNA sequence	gi|21414022	20.7/4.71	369	38	9	3.2e-032
12	PREDICTED: keratin, type II cytoskeletal 1 *[Gorilla gorilla gorilla]*	gi|160961491	66.0/8.15	232	36	12	1.4e-016
13	serine protease	gi|168805933	48.7/6.33	145	9	2	1.1e-010
14	serine proteinase	gi|13641204	48.7/6.33	116	12	5	8.4e-008
15	deoxyribonuclease II family protein	gi|339241449	38.1/5.95	312	30	9	2.1e-027
16	53 kDa excretory/secretory antigen	gi|805126	4.71/8.42	280	21	8	3.3e-024
17	conserved hypothetical protein	gi|339258426	13.5/4.54	121	19	2	2.6e-008
18	Not identified	-	-	-	-	-	-
19	conserved hypothetical protein	gi|339258426	13.7/4.54	209	29	3	4.2e-017
20	conserved hypothetical protein	gi|339258426	13.5/4.54/	205	42	4	1.1e-016
21	conserved hypothetical protein	gi|316966355	13.5/4.54	92	30	2	2.2e-005
22	conserved hypothetical protein	gi|316966355	13.5/4.54	70	19	2	0.0035
23	conserved hypothetical protein	gi|339258426	13.5/4.54	231	29	3	2.6e-019
24	conserved hypothetical protein	gi|339258426	13.5/4.54	254	59	5	1.3e-021
25	conserved hypothetical protein	gi|339258426	13.54.54	248	59	5	5.3e-021
26	Not identified	-	-	-	-	-	-
27	MBTsMLA019T7SEQ *T. spiralis* muscle stage larvae (BC) *T. spiralis* cDNA clone MBTsMLA019 5′, mRNA sequence	gi|13198997	19.9/4.76	345	38	6	8.1e-030
28	MBTsMLA019T7SEQ *T. spiralis* muscle stage larvae (BC) *T. spiralis* cDNA clone MBTsMLA019 5′, mRNA sequence	gi|13198997	19.9/4.76	359	38	6	3.2e-031
29	Not identified	-	-	-	-	-	-
30	Not identified	-	-	-	-	-	-
31	MBTsMLA019T7SEQ *T. spiralis* muscle stage larvae (BC) *T. spiralis* cDNA clone MBTsMLA019 5′, mRNA sequence	gi|13198997	19.9/4.76	423	55	6	1.3e-037
32	TPAF-aac52c09.g1 *T. spiralis*_EST *T. spiralis* cDNA 5′, mRNA sequence	gi|157957485	21.6/9.48	382	57	7	1.6e-033
33	ps19d01.y1 *T. spiralis* ML CMVsport jasmer *T. spiralis* cDNA 5′, mRNA sequence	gi|148310851	17.7/10.32	223	53	6	1.3e-017

^a^Theoretical molecular mass (kDa) and isoelectric point (pI).

### Functional categorization of surface proteins by gene ontology

**Figure 4:**
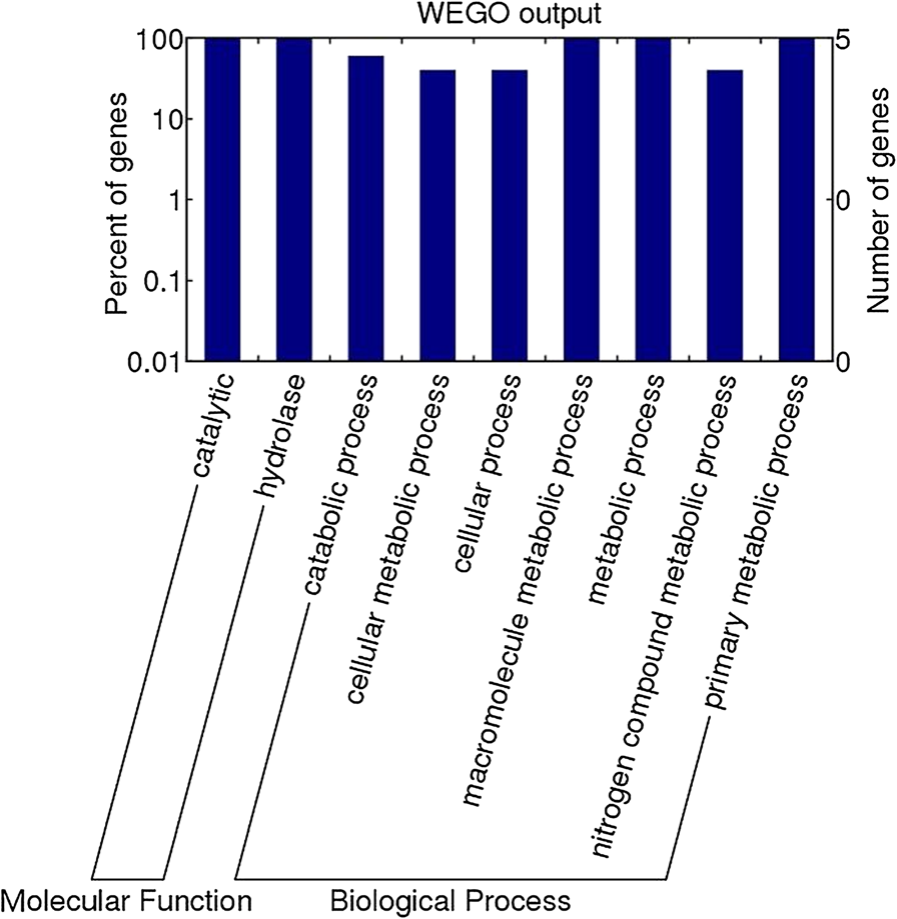
**GO categories of *T. spiralis *muscle larval surface proteins.** The proteins were classified into molecular function and biological process by WEGO according to their GO signatures. The number of genes denotes that of proteins with GO annotations.

Gene Ontology (GO) signatures of 5 (partial P49 antigen, deoxyribonuclease II family protein, serine protease [gi|168805931], serine protease [gi|168805933], and serine proteinase) out of the 14 proteins identified were available. To further understand the functions of the proteins identified in this study, we queried against the InterPro databases and those resultant proteins were classified into molecular function and biological process according to GO hierarchy using WEGO (Figure 4).

For the molecular function ontology, the classification results showed that all the above- mentioned five *Trichinella* genes were annotated with catalytic activity (GO: 0003824). Catalytic activity specifically refers to hydrolase activity (GO: 0016787).

In the biological process category, five proteins of *T. spiralis* were related to metabolic process (GO: 0008152, 5 proteins, 100% of 5 annotated peptides) and cellular process (GO: 0009987, 2, 40%). Most of the assigned metabolic process could be assigned to nitrogen compound metabolic process (GO: 0006807), catabolic process (GO: 0009056), macromolecule metabolic process (GO: 0043170), cellular metabolic process (GO: 0044237), primary metabolic process (GO: 0044238). The proteins in the cellular process group are related to cellular metabolic process (GO: 0044237). Most of the cellular and metabolic processes were related to synthesis and degradation of macromolecules, particularly carbohydrates, nucleotides and proteins, which might be associated with the invasion and development of *T. spiralis* infective larvae.

## Discussion

The cuticle surface of *T. spiralis* muscle larvae is directly exposed to the host’s immune system and present key target antigens that induce the host immune responses. As such, the surface antigens may play an important role in the invasion, immune evasion of the larvae and mutual adaptation between parasites and host immune response [29]. The surface proteins include a group of proteins that can signal various biological processes, including immune reactions, adhesion molecules, and enzymes. In this study, the IFT results showed that the surface proteins-stripped larvae were not recognized by sera of immunized mice with surface antigens, indicating that surface proteins of muscle larvae were successfully stripped and prepared. The results from SDS-PAGE showed that surface and ES proteins had the same 7 protein bands, and surface and somatic proteins also had the same 7 protein bands, suggesting that the partial surface proteins might derive from the ES proteins which were incorporated on the cuticle [7]. Western blotting analysis showed that seven protein bands of the surface proteins were recognized by mouse infection sera at 18 dpi, demonstrating the surface proteins might be used as early diagnostic antigens for trichinellosis.

*T. spiralis* infective larvae do not possess oral appendages or a spike [30], implying that the invasion of intestinal epithelial cells may not be simply a result of mechanical penetration but may be mediated by surface proteins and the oral secretions of the infective larvae [31]. The larval surface proteins may interact with intestinal epithelial cells and may play a key role during the larval invasion of intestinal epithelial cells*.* However, the specific protein molecules related with the larval invasion of enterocytes in *T. spiralis* surface proteins have not been identified [11].

In this study, our results demonstrated a protein profile of the *T. spiralis* surface proteins migrating as shown in Figure 3. A total of 33 protein spots were selected and identified by MALDI-TOF MS. Of these, 27 protein spots were identified, which represented 15 different proteins. Fourteen out of 15 different proteins were identified as *T. spiralis* proteins. Out of the 27 successfully identified protein spots, five spots (4, 6, 7, 9 and 15) were identified as deoxyribonuclease II family protein, and they have the same MW and pI. Six spots (17, 19, 20, 23, 24, and 25) were identified as a conserved hypothetical protein, and they have the same pI but small differences in MW. Two spots (21 and 22) were identified as a conserved hypothetical protein, and they have the same MW and pI. Three spots (27, 28, and 31) were identified as MBTsMLA019T7SEQ *T. spiralis* muscle stage larvae (BC) *T. spiralis* cDNA clone MBTsMLA019 5′, mRNA sequence. In comparison, they have the same MW and pI. A previous study has also demonstrated that *T. spiralis* may express more than one isoforms of the protein and that a common precursor protein could undergo variable post-translational processing [16,17,32]. These modifications could be related to phosphorylation or acetylation of the proteins after translation, and they could be vital for the protein’s biological functions, such as parasite survival, immune escape and immunopathogenesis. There is also a possibility that these proteins are members of the same protein family which share functional domains. Six protein spots (2, 8, 18, 26, 29 and 30) failed to match the proteins to *T. spiralis* ESTs or any sequence of other species of the genus *Trichinella*, which may be due to the low concentrations of the proteins, which therefore failed to produce high quality mass spectrometric data. It is also possibly because the proteins of the six spots were not included in the databases and these proteins have not yet been described.

In order to provide a comprehensive understanding of the roles of *T. spiralis* surface proteins, the proteins identified were functionally categorized based on the GO annotation of biological process and molecular functions. Accordingly, the proteins identified by MALDI-TOF MS might demonstrate their importance and contribution in the process of larval invasion and immune evasion. The classification results of the 14 *Trichinella* genes showed that five genes were annotated with putative molecular functions. All the five *T. spiralis* proteins (partial P49 antigen, deoxyribonuclease II family protein, two serine proteases, and serine proteinase) encoded by these genes have hydrolase activity. The results suggested that the larval invasion of intestinal epithelial cells was possibly mediated by these hydrolase in the larval surface proteins [31,33]. Out of the 14 different *T. spiralis* proteins identified in this study, 9 proteins had no assigned GO terms in the GO database. This is partially due to the limitation of the coverage of the current GO annotation system, and also due to some novel proteins previously described only as putative open reading frames (ORFs).

The partial P49 antigen of *T. spiralis* has been cloned, characterized, and expressed in *Escherichia coli* by recombinant DNA methods [34]. The recombinant P49 is a potentially valuable antigen both for vaccine development and immunodiagnosis. The deoxyribonuclease II family protein is known to be a lysosomal enzyme, introduce single and double-stranded breaks into supercoiled plasmids in the presence of EDTA, and mediate internucleosomal DNA digestion characteristic of apoptosis following intracellular acidification [35,36]. Serine proteases are important in a wide variety of biological processes, including digestion, blood coagulation and fibrinolysis. They are enzymes that cleave peptide bonds in proteins, in which serine serves as the nucleophilic amino acid at the enzyme’s active site [37]. In parasites, serine proteases are known to be involved in the invasion of host tissues and cells [38], and in nematodes are likely to be important in molting. Several secreted serine proteases have been identified in *T. spiralis* ES proteins, including the trypsin-like 45 kDa antigen and the serine protease TspSP-1 [39,40]. Our previous studies showed that when *T. spiralis* muscle larvae were activated by bile and co-cultured with intestinal epithelial cells, the transcription and expression level of a serine protease gene was obviously up-regulated, compared with the untreated normal muscle larvae [41-43]. The results of the present study further suggested that the serine proteases might be related with the larval invasion of intestinal epithelial cells, which is needed to be confirmed in further experiments.

## Conclusions

This study showed that *T. spiralis* muscle larval surface proteins had a total of approximately 33 proteins spots with pI 4–7 and MW 10–66 kDa. Out of the 14 proteins identified as *T. spiralis* proteins, 5 proteins (partial P49 antigen, deoxyribonuclease II family protein, two serine proteases, and serine proteinase) had catalytic and hydrolase activity. These *T. spiralis* surface proteins identified might be invasion-related proteins, early diagnostic antigens for trichinellosis and targets for a vaccine.

## Competing interests

The authors declare that they have no competing interests.

## Authors’ contributions

JC, ZQW and MYL conceived and designed the experiments. JC, RDL, LW, XZ, and PJ performed the experiments. JC RDL, MYL, and ZQW analyzed the data and wrote the manuscript. All authors read and approved the final version of the manuscript.
